# Nanotechnological Strategies for Protein Delivery

**DOI:** 10.3390/molecules23051008

**Published:** 2018-04-25

**Authors:** María Rocío Villegas, Alejandro Baeza, María Vallet-Regí

**Affiliations:** 1 Departamento de Química en Ciencias Farmacéuticas, Facultad de Farmacia, UCM, 28040 Madrid, Spain; mr.villegas@ucm.es; 2 Centro de Investigación Biomédica en Red de Bioingeniería, Biomateriales y Nanomedicina (CIBER-BBN), 28029 Madrid, Spain

**Keywords:** protein delivery system, PEGylation, liposomes, nanoparticles, polymeric nanocapsules, protein therapy

## Abstract

The use of therapeutic proteins plays a fundamental role in the treatment of numerous diseases. The low physico-chemical stability of proteins in physiological conditions put their function at risk in the human body until they reach their target. Moreover, several proteins are unable to cross the cell membrane. All these facts strongly hinder their therapeutic effect. Nanomedicine has emerged as a powerful tool which can provide solutions to solve these limitations and improve the efficacy of treatments based on protein administration. This review discusses the advantages and limitations of different types of strategies employed for protein delivery, such as PEGylation, transport within liposomes or inorganic nanoparticles or their in situ encapsulation.

## 1. Introduction

The transition of a prebiotic to a biotic Earth was determined by the appearance of self-sustained, self-replicating and self-assembled life. Living organisms are complex bioreactor systems, where numerous biochemical reactions occur simultaneously, allowing their structures to self-assemble, replicate itself and be transmitted. One example of structures that self-assemble with precision and fidelity are proteins. All human cells have the same genetic information, which is contained in its deoxyribonucleic acid (DNA) and encodes proteins.

Proteins are aminoacid strands which are folded in characteristic three-dimensional structures determined by the aminoacid sequence and the microenvironment. These chains form a secondary structure characterized by alpha helices and beta sheets stabilized by intramolecular hydrogen bonds. The secondary structure is then folded into a tertiary structure governed by hydrophobic and hydrophilic interactions so that hydrophobic zones of the protein are in its core and the hydrophilic parts remain exposed to the aqueous medium on the protein surface. The highly specific structures produced by protein folding and their precise amino acid sequences determine protein function. The huge variety of highly-specific chemical processes needed for life is obtained by the great versatility of potential protein structures and conformations.

Proteins performed essential functions, such as catalyzing biochemical reactions [1], signal transduction [2], defensive functions [3], regulatory functions [4,5], controlling cell fates [6], providing cellular and tissue structure [7,8], as molecule carriers [9,10,11], and maintaining a fine balance between cell survival and programmed death. For this reason, proteins are called the “engines of life”.

Eukaryotic cells contain thousands of proteins that participate in the normal cellular function [12]. Their correct function is vital to maintain homeostasis in the body. Protein dysfunction is related to numerous diseases such as diabetes, which consists of unbalanced regulation of insulin, hemophilia which is a defect in coagulation protein levels, neurological disorders (Alzheimer’s [13] and Parkinson’s disease [14]), cystic fibrosis which is related to a defective folding and export of proteins from the endoplasmic reticulum [15] and cancer (about 50% of all human tumors have a mutant p53 protein) among others [16].

Consequently, the use of proteins as therapeutic molecules appears as an attractive and promising therapy for cancer [17], autoimmunity/inflammation [18], infection [19] and genetic disorders and it has shown high efficacy for the treatment of numerous diseases [20,21]. Protein therapeutics include antibodies, cytokines, transcription factors and enzymes, among others.

Although proteins hold great potential as therapeutics, their clinical application is limited due to their labile nature. The functional conformation of a protein is only slightly more stable (5–20 kcal/mol in free energy) than unfolded conformations (the term unfolded is used to refer to any non-functional conformation resulting from an unfolding process). This means that the process of protein folding until functional conformation presents a negative increment of Gibbs free energy [22,23,24,25]. From an entropic point of view, this is an unfavorable process. Entropy acts at a local level, that involves translational, rotational and vibrational degrees of liberty at the molecule scale, and non-local level, which include volume and chain configurational freedom [23]. The resulting negative increment of Gibbs free energy is due to a negative increment of enthalpy in the folding process. The forces that stabilize the structure are hydrophobic interactions, electrostatic forces, local peptide interactions, hydrogen bonding and Van der Waals forces [23]. The balance between forces that mantain the functional three-dimensional structure of folding is fragile and can be destabilized under small changes in temperature, pH, type and salts concentration, concentration of serum proteases, solvent or external microenvironment can induce protein unfolding leading to biologically inactive conformations [22].

Moreover, proteins can suffer from proteolysis by proteases present in the bloodstream and in living tissues, which induce an irreversible change in their structure and, therefore, a loss of biological function. In addition to the low stability of proteins, protein delivery presents additional problems. Foreign proteins administered intravenously can often be recognized by opsonins and many scavenger receptors. Opsonization with lipoproteins results in their accumulation in hepatocytes and other tissues rich in lipoprotein receptors. Moreover, attachment of complement proteins leads to an immediate clearance from the bloodstream by macrophages which form the macrophage phagocytic system (MPS) [26,27]. Thus, administered proteins are often cleared out rapidly by spleen, liver or kidney, where they can be accumulated undesirably and activate immune responses.

Clinical uses of proteins are limited by their low stability [22] against temperature, solvent changes, changes in pH, serum proteases, freezing cycles and storage. In addition, proteins are usually unable to cross cell membranes [28], they can activate immune responses and be accumulated in tissues and they show fast clearance after intravenous administration [29]. All of these problems have given rise to the development of recombinant proteins, which try to palliate these limitations. Recombinant insulin was the first commercially available recombinant protein approved by the US FDA in 1982 [30,31,32,33,34]. In recent years, between 2011–2016 the Food and Drug Administration Center for Drug Evaluation and Review (CDER) and the Center for Biologics Evaluation and Review (CBER) have approved 62 therapeutic proteins [35].

**Figure 1:**
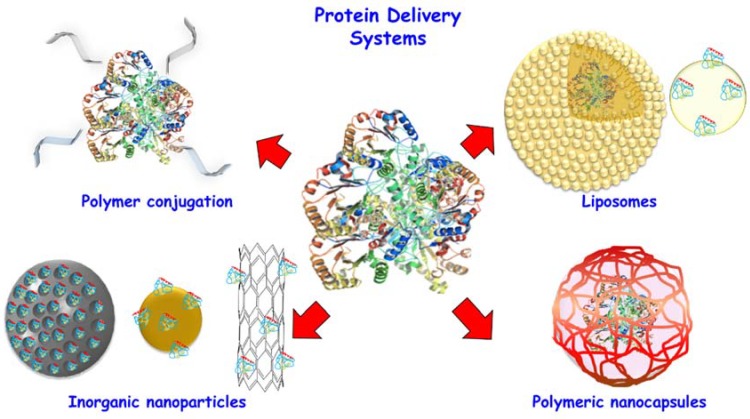
Scheme of different protein delivery systems.

Therapeutic proteins represent a fast-growing proportion of marketed drugs. Nevertheless, such “biodrugs” have important hurdles that hamper their application. As mentioned above, proteins exhibit unfavorable intrinsic properties. Nanomedicine has emerged as powerful tool to try to overcome these strong limitations. The scientific community have done vast efforts to design versatile, protective and functional protein delivery systems to achieve an improved therapeutic effect. Important efforts in this area had been focused in the conjugation of proteins with polymeric chains in order to increase their size and block recognition zones. Another strategy has been their transport attached onto the surface of nanocarriers or inside their structure, which has often been employed to allow proteins to enter inside cells. As another option, proteins have been entrapped in the aqueous core of lipidic vesicles for prolonging their circulation time within the blood stream. However, in the ultimate years, nanomedicine has centered its efforts in the development of encapsulation methodology that allows wrapping of proteins into polymeric nanocapsules. This class of materials allow design of robust and flexible protein-coating and engineer it as non-degradable or degradable in function of the biomedical application. Some of the most important approaches are collected in this review and shown schematically in Figure 1.

## 2. PEGylation

The most common strategy to increase the circulation times of protein in the bloodstream is the covalent conjugation of polymers on the protein surface, being the most popular poly(ethylene glycol) (PEG). Protein conjugation with PEG is often achieved by its reaction with free amine groups from lysine residues on the protein surface [36]. PEG is a polymer approved by FDA as “generally recognized as safe” [37] and is known to be able to coordinate with water molecules that create a hydrophilic corona around the protein via hydrogen bonding. As a result of this hydration layer and the high flexibility of the PEG chains, PEGylation increases the hydrodynamic radius of the PEGylated protein facilitating its solubility and hindering its renal clearance [38,39]. PEGylation produces a steric impediment to opsonization, improving the plasma half-life of proteins and avoiding the clearance by MPS [40,41,42]. PEG acts as a hindrance to proteases and therefore, make the proteins more resistant to proteolytic degradation. In this way, PEGylation can preserve the protein structure and therefore, its function. Moreover, PEGylation can hide antigenic zones of foreign proteins which avoids the formation of specific antibodies against them decreasing their immunogenicity. The first evidence of the advantages of protein PEGylation was reported by Abuchowski et al. in 1977 [43,44]. Later, in 1990, adenosine deaminase was the first PEGylated protein approved by FDA and commercialized for the treatment of immunodeficiency diseases [45]. Since then, different conjugates have been approved [46] and marketed such as, for example, PEGylated interferon α2b y α2a for the treatment of hepatitis C [47], granulated stimulating factor for treatment of neutrophenia caused by chemotherapy [48,49], and epotein-β protein for the treatment of chronic renal failure [50], among others.

Unfortunately, it has been found that approximately 25% of patients present or develop anti-PEG antibodies previously or immediately after the first administration of PEG-protein conjugates [42,51,52]. This fact implies the rapid clearance from blood of the administered proteins and nullifies their efficacy for systemic treatments.

Another hydrophilic polymer used to prepare enzyme conjugates is poly(vinylpyrolidone) (PVP). After its use as plasma expander during Second War World [53], PVP was the first polymer reported to form polymer-drug conjugates [54]. PVP is considered a harmless compound [55] and it also improves the circulation time of enzymes [56]. Unfortunately, PVP conjugates can increase antigenicity compared to free enzymes, as is the case for uricase [57]. As an alternative to hydrophilic polymers, zwitterionic polymers have also been used for their capacity to coordinate water molecules via hydrogen bonding. Zwitterionic polymer conjugates have shown similar pharmacokinetic profiles to PEGylated systems [58]. Another option is the conjugation with dextran, which is a polysaccharide that also extends the circulation time in blood [59]. However, intravenous administration of dextran can produce life-threatening anaphylaxis [60,61].

Since polymer conjugation is a simple and popular method, it is employed for many commercial formulations. However, polymer conjugation often blocks the active sites of enzymes. Thus, the enzyme partially or completely loses its catalytic activity [62]. This fact, along with those above mentioned, constitute strong limitations of polymer-protein conjugates.

As an example, of commercial method to protein delivery are the so called chariot systems. These consist of a 2843 Da peptide able to form a non-covalent complex with the protein that allows the transport of biologically active proteins into the cells with an efficiency of 60–95% [63].

## 3. Liposomes

One interesting approach for protein delivery involves their transport in liposomes. Liposomes consist of concentric lipid bilayer vesicles surrounding aqueous compartments. The vesicles are formed by phospholipids and their structure is similar to that of the cellular membrane. Proteins can be carried inside the aqueous core of the liposome or can be grafted on the lipid surface.

The protein functionalized liposome techniques include loading the liposome into the core and on the surface. As examples, Szoka et al. [64] reported a technical procedure to prepare liposomes with large aqueous spaces. This method allowed them to encapsulate water-soluble materials, such as proteins, with high efficiency. This process consists in the formation of lipid vesicles when aqueous buffer was introduced into a mixture of phospholipids in organic solvents. The authors reported that, in spite of the fact the organic solvent produced protein denaturalization, an appreciable amount of activity (41%) was retained. On the other hand, loading of proteins on the liposome surface have been widely reported. Shao et al. [65] synthesized liposomes including a porphyin-phospholipid. This phospholipid, which is able to chelate cobalt, allowed the effective capture of His-tagged proteins As an additional example, Blenke et al. [66] develop liposomes able to conjugate azide-protein by “click chemistry” which allows more control at the conjugation site than commonly used coupling chemistries.

The cell-like nature of lipid systems eases the translocation of the protein carried into the cytoplasm or lysosomes inside the cells. Uptake of liposomes by cells is produced by an initial liposome adsorbtion onto the cell membrane, followed by an endocytosis step [67]. Liposomes possess excellent characteristics for protein delivery, including biocompatibility, the capacity to maintain an aqueous environment around the protein and modulable size and charge which can be obtained by selection of phospholipids [68,69]. The composition of the phospholipids that form the liposomes determines their characteristics. For example, cationic liposomes facilitate the adsorption and endocytosis processes by interaction with negatively-charged phospholipids on the cell membrane. Moreover, they disrupt the enodosome by the proton sponge effect allowing the cargo release to the cytosol [70]. As an example, cationic liposomes were used to carry β-galactosidase and caspases [71]. However cationic liposomes present low stability in the presence of serum proteins and poor stability in vivo [72]. Also, these systems produce a certain toxicity due to apoptosis induced by their cationic moieties [73]. Neutral liposomes have demonstrated a good ability to enter into the cell, but the protein cargo is often trapped within lysosomes and, therefore, the transported proteins are digested in the lysosome due to their inability to escape from endosomes.

Additionally, a potential problem of liposomes as protein carriers is their rapid clearance by the MPS [74]. This problem can be partially palliated by conjugation with PEG chains on the lipid surface. In fact, liposome PEGylation has been shown to extend their circulation half-life from 30 min to 5 h [75]. This long circulation time is due to the increase in the hydrodynamic volume of the system and the capacity of PEG to avoid the immune response by steric impediment. However, a high degree of functionalization with PEG produces a reduction in the melting temperature of the liposomes, which entails their destabilization, whereas a low functionalization decreases the achievable circulation time [76]. Moreover, a repeated administration of PEGylated liposomes results in an accelerated blood clearance [77]. In addition, the nature of liposomes limits their clinical use, since liposomes are not robust and can release the cargo protein when are in an environment with a high concentration of lipoproteins, as is the case in the bloodstream [67,78,79].

In spite of all these limitations, there are commercial kits for protein delivery based on liposomes, as is the case of lipofectamine, which is a cationic liposome able to carried DNA, siRNA and proteins into the cells in a fast, simple, and reproducible method [80].

## 4. Inorganic Nanoparticles

Inorganic nanoparticles have been explored as interesting nanodevices due to the fact they are robust and easily modulable. The proteins can be carried on the nanocarrier surface or inside their structures, in the case of porous nanoparticles. The applications of such materials however, require chemical and/or biological modification to satisfy the requirements for cellular delivery, such as biocompatibility and long circulation times [81].

### 4.1. Mesoporous Silica Nanoparticles

An example of inorganic nanoparticles is mesoporous silica nanoparticles (MSN). These nanoparticles have also been investigated as protein carriers. They are characterized by a high surface area and tunable pore size [82], that provide a high cargo-loading capacity and allow delivering a wide variety of proteins [83]. Tu and coworkers [84] have recently proposed this type of nanoparticles to deliver proteins with different molecular weights (12.4–250 kDa), size (2.3 × 2.6 × 4 to 7 × 8 × 10 nm) and isoelectric points (4.5–11.35). Functionalization or not of the pores of nanoparticles with amine groups favors the electrostatic interaction with proteins bearing negatively or positively charged groups. Moreover, their surface can be easily modified with nickel moieties to chelate polyhistidine-tagged proteasomes. Cells treated with exogenous proteasomes are able to significantly degrade tau aggregates, a pathological hallmark of Alzheimer´s disease, compared to the free proteasomes [85]. These nanoparticles have improved the intracellular delivery of membrane-impermeable proteins [83]. As example, MSNs demonstrated be able to intracellularly deliver cytochrome C in human cervical cancer cells (HeLa), an induce a significant cell death [86,87].

### 4.2. Gold Nanoparticles

**Figure 2:**
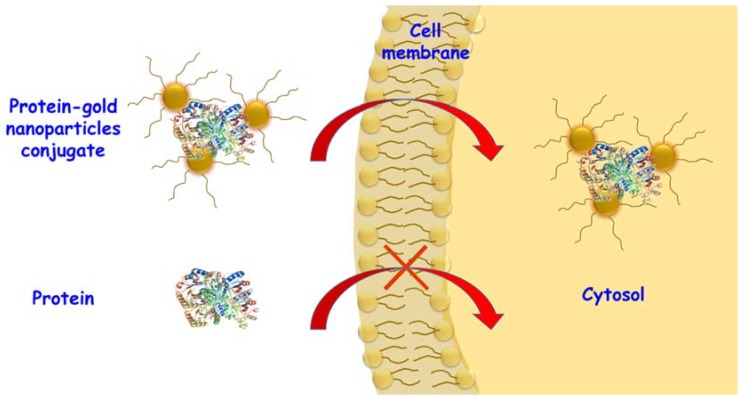
Scheme of protein adsorbed onto peptide coated gold nanoparticles to improve cell internalization by endocytic pathway.

Gold nanoparticles have been studied as protein delivery systems for their unique optical properties, low toxicity, bio-inertness and easily funcionalizable surface via thiol groups [88,89,90,91,92]. The first intracellular protein delivery system using gold nanoparticles was reported in 2004 [93]. In that work, BSA was previously modified with CPP and was adsorbed onto gold nanoparticles. Protein adsorption on the surface of gold nanoparticles allowed its delivery into the cell cytoplasm, followed by nuclear localization mediated by CPP. Gold nanoparticles have been functionalized with positively-charged moieties which allow the adsorption of anionic proteins via electrostatic interactions. Additionally, the adsorption on the gold surface inhibits the protein activity until it is released in the cytosol as consequence of its exposure to the glutathione present in this environment [94]. As an additional example, gold nanocarriers were also studied to carry vascular endothelial growth factor (VEGF) [95]. VEGF was attached on the gold nanoparticle surface via the thiol groups of cysteine residues in VEGF. The conjugation with VEGF produced a recovery of blood perfusion to values of normal tissue in hind-limb ischemia mice, as opposed to administration of free VEGF that did not produce significant changes compared to the control group. β-Galactosidase was also adsorbed in its activated form onto gold nanoparticles coated with short peptide in order to improve its cell internalization presumably via an endocytic pathway (Figure 2) [28].

### 4.3. Carbon Nanotubes

Carbon nanotubes are rolled sheets of graphene formed from sp^2^-hybridized carbon atoms. This class of materials is used for biomedical imaging due to their unique near-infrared (NIR) photoluminescence. Moreover, carbon nanotubes have been explored to shuttle different molecular cargos inside of cells, including short peptides, nucleic acids and proteins [96,97,98,99,100,101,102,103]. Carbon nanotubes are also used as protein delivery systems and have demonstrated to be a polyvalent system able to adsorb a widely variety of proteins on its surface, such as streptavidin, BSA, protein A and cytochrome C for their intracellular delivery by adherent (HeLa and NIH-3T3) and no adherent cell lines (HL60 and Jurkat) [96]. As an example, fluorescent carbon nanotubes were biotinated and subsequently incubated with streptavidin. The carbon nanotube conjugate allowed the intracellular delivery of streptavidin, which is well known for its inability to cross the cell membrane, and induce a dose-dependent effect in HL60 cells.

## 5. Polymeric Nanocapsules

While traditionally protein delivery has been addressed by their conjugation with polymers or delivery in inorganic nanoparticles or liposomes, a new approach has recently emerged. This new strategy consists of the in situ formation of polymeric coatings around the protein (nanocapsules). These nanodevices have demonstrated promising characteristics compared to traditional systems since they combine the flexibility and cell-like properties of liposomes with the sturdiness of inorganic nanoparticles. As will be discussed in the following sections, the polymeric matrix serves as a protective shield and are highly adaptable to the objective pursued.

**Figure 3:**
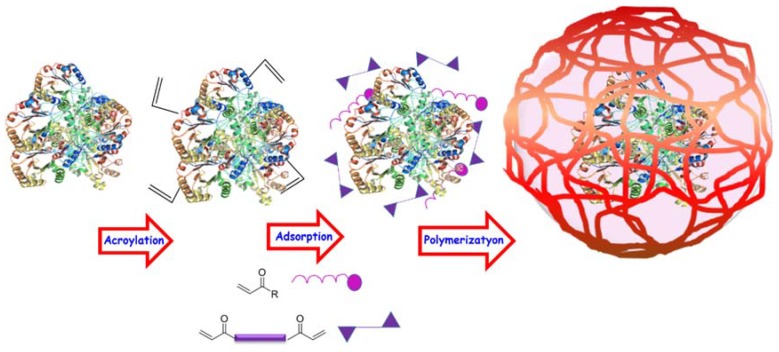
Scheme of the encapsulation process.

Typically, the encapsulation process comprises three steps (Figure 3). Firstly, the protein is functionalized with acroyl groups in order to introduce polymerizable groups on the protein surface facilitating the polymer coating around the macromolecule. This process is usually carried out by the addition of *N*-acryloxysuccinimide (NAS), which can react with amino groups of the lysine residues and the terminal NH_2_ of the protein. Secondly, the acroylated protein is mixed with the monomers in a deoxygenated buffer. The reason for the use of buffers without oxygen is that the presence of this molecule stops the polymerization due to the radical scavenger behavior of triplet oxygen. In this step, the monomers are adsorbed on the protein surface by electrostatic interactions forming a dynamic monomer layer around it. Finally, the third step is the in situ polymerization which is initiated by the addition of radical initiators such as ammonium persulfate (APS) and *N*,*N*,*N*′,*N*′-tetramethylenediamine (TMEDA). APS forms reactive oxygen species in aqueous solution by basic catalysis, and TMEDA acts as the base that allows the process to take place at room temperature [104]. In the case of the formation of stimuli-responsive nanocapsules capable of releasing the housed protein if certain stimuli are present, the acroylation step can be ignored in order to achieve a complete protein departure when the capsule are broken. In order to facilitate the understanding by the reader, the description of the different polymeric nanocapsules will be carried out in two separated groups: non-degradable nanocapsules, which are especially useful for enzyme encapsulation, and degradable nanocapsules suitable for the transportation of therapeutic proteins or enzymes.

### 5.1. Non-Degradable Nanocapsules

This is an appropriate strategy to encapsulate enzymes for which the substrate is a small molecule and the coating is permeable to it. In this sense, the crosslinker responsible for the formation of the polymer matrix must be non-degradable. The most studied non-degradable crosslinker is *N*,*N*′-methylene bisacrylamide (MBA). Using this crosslinker, different types of non-degradable nanocapsules have been developed.

#### 5.1.1. Acrylamide-Based Nanocapsules

Different research groups have studied the use of acrylamide as a structural monomer. This monomer is used in combination with positively-charged and neutral monomers in order to favor the encapsulation process via electrostatic interactions with the protein surface. Lu et al. [105] have developed different nanosystems based on this idea. One of them consisted of the encapsulation of organophosphorus hydrolase (OPH) inside polymeric nanocapsules. Organophosphates are highly toxic and are widely used as pesticides, insecticides and even chemical warfare agents [106]. The use organophosphorus hydrolase (OPH) has been proposed for the destruction of these compounds but, unfortunately, this enzyme has a labile nature. In this work, OPH was coated by a polymeric shell produced using acrylamide (AAm) as a structural monomer, *N*-(3-aminopropyl)methacrylamide hydrochloride (APm) as positively-charged monomer and MBA as crosslinker. As a result of the coating process, the encapsulated enzyme exhibited a higher enzymatic activity with compared with free enzyme in a pH range of 7.5–10. This effect was especially pronounced at a neutral pH. The authors demonstrated that this peculiarity was caused by the basic environment which affect the enzyme when it is inside the capsule. The catalytic activity of OPH is higher under basic conditions and, being APm a basic monomer (pKa = 10) its presence around the enzyme provides a local basic pH which enhances its activity. Moreover, protein encapsulation within these polymeric shells conferred thermal stability, since the free enzyme lost all of its activity after 90 min at 65 °C while the encapsulated enzyme retained 60% of the initial activity under the same conditions. This improvement in thermal stability was demonstrated to be a result of the acroylation process of the enzyme prior to its encapsulation. Nanocapsules obtained after the acroylation process which employed a ratio of NAS:enzyme = 50:1 retained 100% of their enzymatic activity after thermal treatment. Conversely, when the ratio was 10:1, the enzymatic activity after the thermal treatment dropped to 10%. The increase in the thermal stability is the result of the multiple covalent bonds that link the enzyme with the polymeric mesh, which hinder any conformational changes. Also, the encapsulation process improves the enzyme stability in the presence of organic solvents. Encapsulated enzymes retained 50% of their activity in a medium with 30% DMSO in comparison with free enzyme that only showed 10% activity under the same conditions [105]. Polar organic solvents are harmful to proteins because they compete with them in forming hydrogen bonds with water. This leads to changes in their structure resulting in a loss of function. Encapsulation creates a hydrophilic microenvironment around the protein that overcome this limitation. Additionally, enzyme encapsulation eased their storage, since nanocapsules showed 80% activity after five freeze-thaw cycles while free enzymes only maintained 15% of their activity [105]. The encapsulation of OPH in acrylamide-based nanocapsules has also demonstrated to solve the loss of enzymatic activity when embedding or adsorbing enzymes in mesoporous OPH-silica scaffolds [107] and enhanced results obtained with OPH mutants [108] grafted to solid matrices like silica [109], and carbon nanotubes [110].

Encapsulated OPH also showed better results with respect to its naked homolog in in vivo experiments. These experiments consisted of the administration of OPH and OPH nanocapsules in mice that were also administered the organophosphorus compound paraoxon 5 min later. Non-treated mice died 5 min after the paraoxon injection and only two out of three mice with OPH treatment survived, although all of them presented serious toxic effects. Mice treated with encapsulated OPH survived without serious toxic symptoms, validating its therapeutic efficacy.

The encapsulation process has also been studied to coat more than one enzyme so that they can work as an enzymatic tandem [111]. The joint encapsulation of catalase (Cat) and alcohol oxidase (AOx) has demonstrated to decrease alcohol levels in blood in a much more efficient way than the co-administration of the same enzymes encapsulated separately. Moreover, this strategy was demonstrated to be more effective that the use of liposomes loaded with both enzymes. The injection of co-encapsulated AOx and Cat reduced the alcohol levels by 20% in 5 h whereas a liposomal injection did not result in a significant reduction

**Figure 4:**
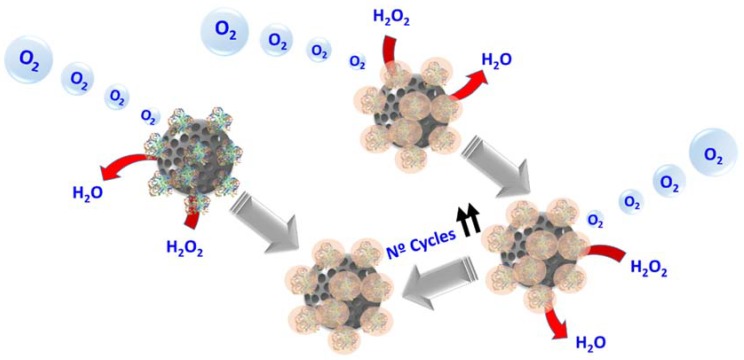
Scheme of nanomotors of mesoporous silica nanoparticles with catalase nanocapsules.

This class of systems have been applied in other less conventional investigation fields such as nanomotors. Nanomotors are self-propelled tiny machines able to perform useful tasks which are normally fueled by the decomposition of peroxide either by platinum or by catalase [112]. In the case of catalase, it has little resistance to oxidation, in such a way, is unable to retain its enzymatic activity during several work cycles. Acrylamide-based encapsulation of catalase has been developed in order to solve this limitation [113]. Catalase capsules demonstrated improved thermal stability of the enzyme and its stability against proteases. After 1 h of incubation at 60 °C the encapsulated enzyme kept more than 50% of its activity whereas its native form only kept 20%. On the other hand, the native enzyme lost all of its activity when it was incubated in the presence of proteases whereas its encapsulated form retained more than 90% of the activity. Additionally, the encapsulation allowed increase the turnover of the enzyme up to more than 10 cycles (Figure 4).

#### 5.1.2. Phosphorylcholine Nanocapsules

One widely used monomer for protein encapsulation is 2-methacryloyloxyethylphosphorylcholine (MPC). MPC has been used as monomer in combination with MBA as a non-degradable crosslinker to obtain a polymeric network of polyMPC (PMPC) by free radical polymerization. This method has been explored to encapsulate horseradish peroxidase (HRP), glucose oxidase (GOx), uricase (UOx) and alcohol oxidase (AOx) among others [114]. These enzymes were previously acroylated with *N*-hydroxysuccinimide ester and subsequently put in contact with the monomers, crosslinker and radical initiators. The formation of the polymeric coating was shown to preserve 75% of the enzymatic activity of the native form and provided them with thermal stability and protection against proteolysis. In this sense, it was proven that the encapsulated UOx retained 85% of its activity after 5 days of incubation at physiological temperature compared to 50% for the native enzyme. In addition, the UOx nanocapsules retained 95% of their activity after 90 min of incubation in a trypsin solution whereas the activity of the native enzyme decreased to 0% after 40 min. These data showed the protective properties of the polymeric layer.

As mentioned in the Introduction, in addition to their protective function, protein delivery systems must be able to avoid the opsonization in serum in order to evade internalization by phagocityc cells, prolonging their plasmatic half-life. PMPC-nanocapsules were demonstrated to fulfill both requirements. Thus, PMPC-nanocapsules were shown to avoid fagocitation by J744A mouse macrophages, with or without previous incubation with mouse serum, while native enzymes were completely uptaken by the macrophages.

Finally, PMPC nanocapsules were shown to evade the adaptive immune system in mice. These data demonstrated the capacity of nanocapsules to avoid the opsonization, phagocytosis and stimulated adaptive immune system. These properties allow a prolonged circulation time of the enzyme and circumvent undesirable accumulation in the liver, kidney and spleen.

In another work it was demonstrated that the capacity of avoiding protein adsorption, which is the main requirement to prolong the circulation time, is due to phosphorylcholine [114,115]. In order to prove this, bovine serum albumin (BSA) encapsulation was done inside four different types of nanocapsules. One of them was synthesized using MPC that resulted in a nanocapsule with a Z potential value of −2.76 mV. Another was synthesized using a combination of AAm and *N*-(3-aminopropyl) methacrylamide (APM) and a last one employing succinic anhydride. This combination allows one to obtain nanocapsules with neutral charge similar to PMP capsules (−2.26 mV), positive charge (+5.28 mV) and negative charge (−15.37 mV), respectively. All of them presented a size of approximately 10 nm during 8 days in phosphate buffer saline (PBS) under physiological conditions. Only positively-charged nanocapsules showed opsonization when they were incubated in the presence of serum proteins and produced a considerable decrease of the cell viability to 75% when they were incubated with HeLa cells at high concentrations (1600 nM). Positively-charged nanocapsules were phagocytosed by J744A.1 mouse macrophages, while neutral and negatively-charged nanocapsules were significantly less engulfed by macrophages and, finally, negligible phagocythosis was found in macrophages incubated with PMPC-nBSA. These results indicated that PMPC was responsible for avoiding endocytosis by macrophages. In vivo studies showed that positively-charged BSA nanocapsules were removed from the bloodstream and accumulated in liver and kidney. Similar results, although no so pronounced, were obtained for negatively-charged capsules. The fast clearance from the bloodstream was caused by interactions between positively-charged nanocapsules and serum proteins, which results in aggregates of proteins being easily withdrawn by liver fenestra and kidney glomeruli. Moreover, highly-charged surfaces (both positively and negatively) activated the complement system, leading to a formation of immune complexes that are easily recognized by Kupffer cells and accumulate in the liver. Neutral acrylamide-BSA nanocapsules showed much slower blood clearance than charged systems. Finally, PMPC-nanocapsules maintain 70% of their enzymatic activity after being in circulation for 8 day.

In regard to pharmacokinetics, it has been studied that PMPC-nanocapsules show an 8-fold increase in their plasmatic circulation time compared to native enzyme ($t_{1/2}=48.92\;h$ vs 6.43 h of native UOx), with clearance rate 169 times lower (11.01 mL/h vs 0.065 mL/h) [116]. Furthermore, the distribution volume of the native enzyme was 22 times higher than for the encapsulated form, what indicates that PMPC nanocapsules stay in the bloodstream whereas the non-encapsulated enzyme invaded the surrounding tissues.

PMPC nanocapsules have also been investigated for the treatment of high urate levels [116]. In order to study their efficacy, native UOx or PMPC-UOx nanocapsules were injected intravenously. The administration of the native enzyme induced a reduction of serum urate from 227 μmol/L to 56 μmol/L in the 5 first hours after the administration. This reduction was followed by an abrupt increase to 293 μmol/L at 24 h, and after that, it slowly decreased to normal values (227 μmol/L) at longer times. The injection of nanocapsules, instead, produced a more sustained and prolonged therapeutic effect, maintaining the serum urate levels below to 150 μmol/L for 5 days. Moreover, repeated administrations of native enzyme induced more immunogenic response, and twice higher levels of white blood cells (WBC) and lymphocytes. PMPC nanocapsules have therefore been proven to be effective for the treatment of hyperoxaluria [29].

Thus PMPC-nanocapsules have been demonstrated to be an extraordinary strategy to encapsulate enzymes. PMPC nanocapsules protect the host enzymes against temperature and proteolysis. Additionally, the polymeric coating prevents their opsonization and phagocytosis by macrophages, allowing longer circulation times and a more sustained and prolonged therapeutic effect.

#### 5.1.3. *N*-Vinylpyrrolidone Nanocapsules

Another type of non-degradable polymeric capsules is based on the use of *N*-vinylpyrrolidone (NVP) as monomer and MBA as crosslinker [117]. Nanocapsules obtained with these monomers display improved thermal, pH and proteolysis stability in comparison to free enzymes. As an example, UOx encapsulated with this monomer maintained 100% of its activity after 3 h whereas the free enzyme retained less than 50%. Also, UOx exhibited activity at pH 4.4–9.4 whereas UOx only had enzymatic activity at physiological pH. With respect to stability against proteolysis, nUOx kept the initial activity after 20 min of incubation with trypsin whereas the native form lost more than 80% of its function after just 5 min. Encapsulation of UOx also avoided the uptake by macrophages, decreasing the internalization percentage from 98% after 1 h (native UOx) to less the 20% after 5 h (encapsulated UOx). Regarding its pharmacokinetic profile, free enzyme clearance rate was 25 times faster than its encapsulated form, with twice the distribution volume. PNVP nanocapsules were evaluated for the treatment of high urate levels. UOx reduced the serum uric acid from normal levels (58.5 μmol/L) to 25.7 μmol/L, but after that, there was a rapid increase up to 91.95 μmol/L after 72 h, followed by a slow reduction to normal levels during 18 days. Administration of encapsulated UOx resulted in constant level of 20 μmol/L during the first 5 days followed by a progressive increase to normal levels, reached after 10 days. Finally, polymer excretion was also studied, showing that 90% of the administrated dose was recovered in urine after 24 h.

### 5.2. Degradable Nanocapsules

**Figure 5:**
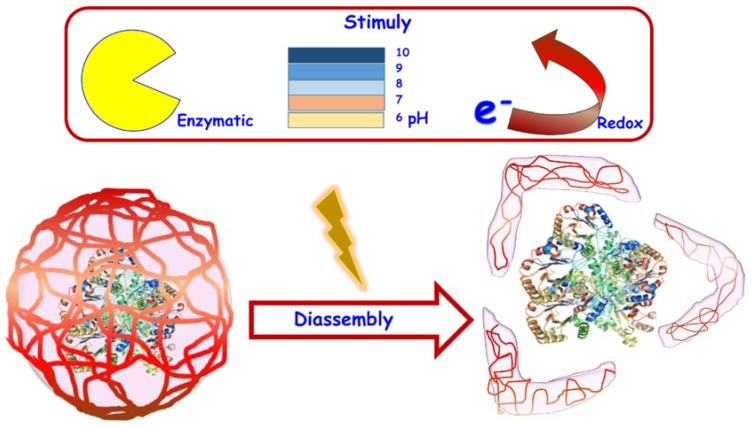
“Stimulation” and “diassembly” of degradable nanocapsules.

When the substrate on which the enzyme acts is so large that the polymeric mesh is not permeable to it or the transportation of a therapeutic protein is intended, nanocapsules must be designed in order to release the host protein when certain stimuli is present (Figure 5). Thus, degradable nanocapsules allow an on-demand release of the housed protein and, therefore, a precise control of the protein administration. Different stimuli have been explored to trigger the nanocapsules disassembly as a function of the pursued purpose and therefore, different crosslinker have been explored. In all of this type of systems, acrylamide (AAm) was employed as structural monomer in combination with other charged monomer to facilitate the absorption of monomers on the surface of the protein or provide anchor points to the resulting capsules.

#### 5.2.1. pH-Respondive Nanocapsules

One widely used strategy is the design of pH-responsive nanocapsules. Acidic conditions are present in different pathologies as solid tumors, which present a mild-acidic environment as a consequence of their accelerated metabolism and hypoxic conditions [118]. Therefore, it is possible to release the enzyme once it has reached the tumor tissue providing pH-responsiveness to the polymeric coating.

**Figure 6:**
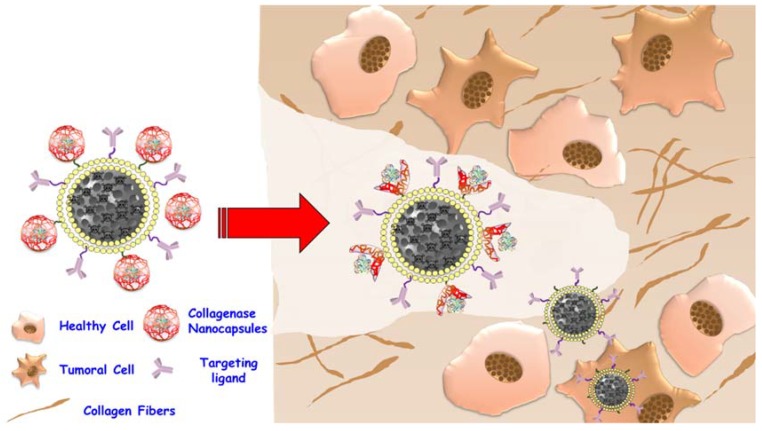
Collagenase nanocapsules to improve the penetration of drug loaded nanocarriers inside tumors.

An interesting use of the proteins in nanomedicine consist of degrade the extracellular matrix allowing the drug loaded nanocarrier to homogenous distribution inside tumoral mass. Tumoral mass often present denser extracellular matrix than healthy tumors. This fact hinders the penetration of nanodevices and, therefore, limits the therapeutic efficacy of drug carried to a peripheral effect [119]. Proteolytic enzymes have been attached onto nanoparticle surface in order to degrade the tumoral extracellular matrix and allow that the nanocarrier reaches deeper zones of the tumor [120]. However, as have been extensively commented, enzymes have strong limitations. Then, in order to obtain a system able to protect the proteolytic enzyme and provide the nanocarriers to penetration capacity until they reach the tumor zone, acid-degradable nanocapsules of collagenase were developed (Figure 6) [121]. These nanocapsules were formed using ethyleneglycol dimethacrylate (EGDMA) as the pH-degradable crosslinker. The enzymatic activity was blocked as a consequence of the encapsulation process until exposed to an acidic environment, where it exhibited its activity. This effect is due to the polymeric mesh providing a steric impediment for the enzyme to act on its substrate, but once the mesh is degraded in the presence of the stimulus, the enzyme is exposed.

Besides pH-responsiveness, the authors investigated the protective function of the polymeric coating against the proteases which are present in the bloodstream. Nanocapsules and free enzyme were exposed to proteases, being observed that the encapsulated enzyme retained 100% of its activity after 3 h whereas the catalytic activity of the free enzyme was reduced by more than 50%.

On the other hand, it is well known that endosomes present acid pH values. Thus, a pH-responsive polymeric mesh can be degraded in the endosomes, upon intracellular entrance, releasing the protein into the cytoplasm. This strategy has been employed in order to avoid the problem of degradation of proteins in lysosomes. In a recent study, pH-sensitive protein nanocapsules have been demonstrated to escape from the endosomes [122]. In this case, glycerol dimethacrylate was employed as pH-responsive crosslinker. The endosomal escape was investigated by the incubation of rhodamine-labelled HRP nanocapsules with HeLa cells and a posterior labeling of early endosomes and late lysosomes. The staining reveals that nanocapsules co-localized with early endosomes and lysosomes at short times, following a gradual release of their cargo inside the cytosol. Also, cells treatment with EGFP nanocapsules was demonstrated to be more effective than treatment with EFGP and TAT-EGFP conjugates. As comparison, protein encapsulation inside liposomes can allow the translocation of the cargo to the cell cytosol, but with lower efficiency [123]. The advantage of pH-responsive nanocapsules over liposomes was shown in a recent publication. In this work, pH-responsive miRNA nanocapsules were developed in order to achieve tumor suppression. The capsules were synthesized using (EGDMA) as the pH-degradable crosslinker. The obtained nanocapsules were able to knock down the expression of mir-21 to 11.5% of the control. This system was demonstrated to be much more effective than miRNA carried inside of liposomes, which only led to a knockdown of 46.2% of control. In addition, nanocapsule endocytosis was five times greater than for the analogous liposomes.

#### 5.2.2. Enzyme Responsive Nanocapsules

Polymeric nanocapsules can be designed to be protease-sensitive. A creative example can be found in the construction of polymeric nanocapsules crosslinked with peptides sensitive to the own loaded enzyme. In this way, the capsule can be degraded from its core by the host enzyme [124]. Caspase 3, an apoptotic protease, was encapsulated using a crosslinker sensitive to this enzyme. The obtained capsule was totally degraded after 200 min at physiological temperature whereas it remained intact at 4 °C, what would enable its storage. This system was shown to induce CP3-mediated apoptosis in HeLa cells in a dose-dependent manner. CP3 cleaves the caspase-activate-deoxyribonuclease inhibitor (ICAD), leading to nucleosome fragmentation and, therefore causing apoptosis. Additionally, in order to allow spatiotemporal control protease release, a degradable crosslinker which contains a photolabile moiety (*o*-nitrobenzyl ester) was incorporated to the nanocapsule composition in such a way that only after ultraviolet exposure the nanocapsule would start to be degraded by CP3 and induced the apoptotic effect. The last example is an ingenious method since it is the own host enzyme which acts to degrade the polymeric nanocapsule, however, in order to deliver another type of proteins, others crosslinker have been explored. A way to obtain nanocapsules with more general and, at the same time, specific mechanism for enzymatic degradation, is to exploit the existence of endoproteases that are expressed in many mammalian cells as triggers. Thus, Tang et al. [125] designed nanocapsules able to specifically degrade by incubation with furin, which is a endoprotease present in several intracellular localizations. Due to is degradability the obtained nanocapsules were able to carry proteins to the cell cytosol or, when the protein was loaded in combination with a localization signal, be localizated in the nucleus. Other proteases have been investigated to trigger the degradation of nanocapsules, such as plasmin and metalloproteinases (MMP) [126]. Plasmin is a serine protease present in the blood and often secreted by tissue cells during new blood vessel formation. On the other hand, MMP are proteases that have an important role in tissue remodeling. Bovine serum albumin (BSA) nanocapsules were obtained using a plamin-cleavable crosslinker (-KNRVK-), or an MMP-cleavable crosslinker (-KLGPAK-) [127]. Specific degradation was studied by incubating the nanocapsules with plasmin or collagenase, obtaining a specific response to the corresponding protease. Another interesting approach for nanocapsules is their use in hydrogels. The formation of hydrogels in the presence of proteins often results in a loss of their structure and activity. Moreover, hydrogels must present big pores in order to achieve a diffusion of nutrients and cell growth. Unfortunately, this also favors a rapid release of the proteins. The encapsulation of proteins and their subsequent addition to hydrogels during consolidation has been demonstrated to be a good strategy to protect the enzyme during hydrogel formation and to avoid protein leaching [126].

Another interesting design is the use of a crosslinker with protease-cleavable specific sequences using aminoacids with different chirality [127]. Peptides formed with l- or d-chiral forms in the same sequence of aminoacids exhibit the same specificity for a determined enzyme but different cleavage kinetics. The rupture kinetic of the sequence NRV formed by d-chiral forms of aminoacids is 10 times slower than the corresponding to the L-form. Using combinations of these crosslinkers, it is possible to achieve different kinetics to obtain nanocapsules with specific and controlled release. This was studied in a recent publication where multiple proteins were encapsulated using this method [127].

#### 5.2.3. Redox-Responsive Nanocapsules

The cell cytosol exhibits lower redox potential than the external intracellular media due to the high concentration of reduced glutathione (GSH). GSH is found in milimolar concentrations inside the cell, whereas it is in the micromolar range in the extracellular medium [128]. Thus, one interesting option is to design nanocapsules able to undergo disassembly exploiting this gradient. Crosslinkers that contain disulfide bonds are redox-sensitive and therefore, their use allow the preparation of nanocapsules that undergo degradation when the system reaches the cell cytosol.

An interesting piece of work reported [129] the synthesis of nanocapsules formed with *N*,*N*′-bis(acryloyl) cystamine as a redox-responsive crosslinker. Capsules designed with the sensitive crosslinker were completely degraded when they were incubated with 2 mM GSH for 2 h at 37 °C. Also, the encapsulation allowed the endocytosis of the protein whereas the native protein was not able to enter the cell. The endocytosed nanocapsules were localized in the early endosomes for 1 to 2 h after incubation, but no co-localization with late endosomes was found, so it was concluded that the protein was released to the cytosol. The treatment of different cell lines with caspase 3 (CP3) in its native form and encapsulated in non-degradable (using MBA as a non degradable crosslinker) or in redox-sensitive nanocapsules demonstrated that only the last system induced apoptosis (which was also dose-dependent). Using the same monomers and crosslinker, Tang et al. [130] developed redox-responsive apoptin nanocapsules. Apoptin is a protein able to go to the nucleus and induce p53-independent apoptosis in tumoral cells without affecting healthy cells, due to a tumor-specific phosphorylation of Thr108 that lead to its accumulation. Mice with MCF-7 breast cancers were treated with apoptin capsules, BSA nanocapsules and PBS, and the treatment with apoptin nanocapsules resulted in a reduction of tumor growth rate, demonstrating its therapeutic effect.

p53 plays an important role in tumoral cell sensitivity against radio- and chemotherapy but also promotes their apoptosis. Since approximately the 50% of all human tumors presents mutations in p53 proteins, it is the most frequent mutant gene [131]. Then, transport of non-mutant p53 copies appears as an interesting strategy. Unfortunately, p53 has a tetrameric structure which is prone to aggregation and therefore, to loss of function [132]. On the basis of this fact, Tang et al. [16] designed protein nanocapsules of p53 using *N*,*N*′-methylenebis(acroyl)cystamine as redox-sensible crosslinker. Also, they included *N*-azidodeca(ethyleneglycol) ethylacrylamide in the polymeric composition in order to avoid the non-selective uptake by the cells due to electrostatic interactions between positive charges provided from the positively-charged monomer in the polymeric mesh with negative charges of phospholipids on the cell membrane. The azide groups of the neutral monomer also allowed the anchorage of targeting moieties by orthogonal chemistry in order to achieve selective endocytosis. Nanocapsules were successfully decorated with a specific targeting peptide, LHRH for which receptors are overexpressed in many breast and prostate cancers, in such a way that nanocapsules were selectively endocytated by cells after 12 h of incubation. Moreover, when they had been formed with a degradable croosslinker, p53 was localized in the nuclei inducing cytotoxicity.

Another type of redox sensitive protein nanocapsules was developed by Thayumanavan et al. [133], who used *p*(PEGMA-co-*p*-nitrophenylcarbonate) as a polymer that contains disulfide bonds and ethylenediamine (ED) or tetraethylene oxide bisamine (PEG-bid-amine) as crosslinkers. The polymer was conjugated to the protein surface via reaction between amino groups of lysine residues on the protein surface with *p*-nitrophenylcarbonate (NPC). The remaining NPCs groups were reacted with the respective bis-amine crosslinker, forming a polymeric shell around the protein. It is important to note that the disulfide bonds are in β-position with respect to the carbamate oxygen, in such a way that under reducing environments, as the cell cytosol, the disulfide bonds are broken, which results in carbamate cleavage, releasing the amino groups of the native protein. The most successful encapsulation was achieved with the crosslinker ED which gave rise to an encapsulation efficiency of 64–67% compared to the 5–7% achieved with the bisamine PEG. The obtained capsule preserved the activity of the enzyme against proteases and was only degraded in the presence of dithiothreitol (DTT), which induced a reducing environment, releasing the host enzyme in its functional structure. This system was demonstrated be able to traffic the protein across the cellular membrane and release it in the cytosol.

## 6. Conclusions

Nanomedicine has developed different systems for delivery and protection of proteins. Their conjugation with polymeric chains allows increased circulation times due to an increase in their size but has limited efficacy to protect them against proteases. Other alternatives are their loading inside liposomes or inorganic systems and onto their surface. These strategies have been demonstrated to protect proteins against external agents. However, liposomes have low physicochemical stability in vivo and inorganic nanoparticles are more sturdy systems but often must be functionalized to be biocompatible. The development of platform nanotechnology for protein delivery based on encapsulation for free radical polymerization has been demonstrated be a widely applicable strategy to encapsulate an extensive range of proteins combining the flexibility of liposomes and the sturdiness of inorganic nanoparticles. All these strategies are summarized in Table 1.

As conclusion, several conditions must be fulfilled in order to achieve an efficient protein delivery system: (1) the transported protein must keep its functional structure during the encapsulation process; (2) the carrier must present high loading capacity; (3) the protein should be protected against enzyme degradation, proteolysis or thermic denaturalization, among others; (4) the nanocapsules must allow hide the protein activity until the nanodevice reaches the target site. Moreover, depending of the enzyme work on large or impermeable substrate to the polymeric coating, the enzyme nanocapsule should be designed to dissemble in response to certain stimuli releasing the transported protein with their function intact. Finally, (5) nanocapsules should avoid opsonization and increase the half-life of the enzyme.

As it has been extensively described along this review, polymeric nanocapsules have shown be able to comply all of these requirements by a specific selection of the monomers and crosslinkers that conform to them. This technique has been demonstrated to improve the abovementioned aspects in comparison with liposome encapsulation, polymer protein conjugation and loading in inorganic nanoparticles which have been traditionally employed. Thus, the protein encapsulation strategy has emerged as an approach with high potential to develop the next generation of protein treatments.

## Figures and Tables

**Table 1:** Summary of Protein Delivery Systems.

Strategies for Protein Delivery	
Pegylation	This strategy consists in the conjugation of polymeric chains on protein surface. This allow increase significally the times of circulation of proteins in bloodstream, however has a limited efficacy to protect against proteases attack.
Liposomes	Liposomes are biocompatible and cell-like nanodevices. Proteins can be delivered inside the aqueous core of liposomes or attached on their surface. Liposomes are characterized by high flexibility but their use are limited by small stability in human body.
Inorganic nanoparticles	Mesoporous silica nanoparticles, gold nanoparticles and carbon nanotubes allow the delivery of proteins on their surface or inside them. They are characterized by a high sturdiness but have a poor flexibility.
Polymeric nanocapsules	This strategy consist in a polymerization in situ around the protein making a polymeric coating. This strategy can be used to a large number of proteins and allow the design of nanocapsules both degradable and non-degradable. This class of systems combine the sturdiness of inorganic nanoparticles with flexibility of liposomes
